# Neighborhood Threat of Eviction over Time and Risk of Preterm Birth in Black American Women

**DOI:** 10.1007/s40615-025-02465-y

**Published:** 2025-05-13

**Authors:** Shawnita Sealy-Jefferson, Loretta J. Ross, Kyra Sanders, Karen Harry, Tamika Anderson-Mays, Roquesha Oneal, Cassy Jones-McBryde, JoAnn Booth, Brittney Francis, Benita Jackson, Scarlett Bellamy

**Affiliations:** 1 Ohio State University, 1841 Neil Avenue, Columbus, OH 43210 USA; 2 Smith College, Northampton, MA USA; 3 Social Epidemiology to Combat Unjust Residential Evictions (SECURE) Study Community Advisory Board, Detroit, MI USA; 4 Harvard University, Cambridge, MA USA; 5 Boston University, Boston, MA USA

**Keywords:** Eviction, Preterm birth, Structural racism, Reproductive justice, Neighborhood, Trauma

## Abstract

**Background:**

Black communities are disproportionately impacted by dual crises: residential evictions and adverse birth outcomes. A growing literature has documented the spill-over effects of neighborhood evictions on adverse birth outcomes, but none have examined associations between these exposures over time and risk of preterm birth (PTB) among Black women.

**Methods:**

We linked survey data from the Life-course Influences on Fetal Environments Study (*n* = 807) and publicly available block group-level eviction filing rate data. Addresses from the preconception (from 2007 to 2009) and during pregnancy neighborhoods (from 2009 to 2011) were linked to data from the Eviction Lab. Eviction filing rate trajectories included (1) steady low (referent), (2) steady high, (3) decreasing, and (4) increasing categories. PTB was defined as birth before 37 completed weeks of gestation and was abstracted from participant medical records. Modified Poisson regression with robust error variance estimated relative risk and 95% confidence intervals. Models were adjusted for predictors of residential selection (income, education, marital/cohabiting status, and age), as well as duration of residence in current neighborhood, current neighborhood sociodemographic disadvantage, and residential move from the before pregnancy to during pregnancy neighborhood.

**Results:**

Preterm birth was experienced in 16.2% of the sample (*n* = 131), and the mean age of participants was 27 years. In adjusted models, PTB risk was strongly associated with increasing eviction filing rates (compared to steady low) (relative risk: 1.68, 95% CI: 1.05, 2.68).

**Conclusion:**

Our results provide new evidence about the spillover effects of increased neighborhood threat of eviction over time, on risk of PTB among Black women. Future interventions, including policy solutions aimed at addressing the eviction and PTB crises, are warranted.

**Supplementary Information:**

The online version contains supplementary material available at 10.1007/s40615-025-02465-y.

## Introduction

Preterm birth (PTB), or birth prior to 37 completed weeks of gestation, is a leading cause of infant mortality. Troubling and persistent racial inequities in preterm birth exist in the USA, with Black Americans (hereafter, Black) disproportionately affected [1]. Traditional individual-level risk factors for PTB, such as biology and behaviors, do not account for these racial inequities, though structural racism related exposures [2] including those from racially segregated neighborhood environments [3] likely play a significant role [4].

Neighborhoods are important contextual determinants of inequities in population health, and most existing studies are limited to exposures from the current neighborhood due to limited residential history data [5, 6]. However, theoretical and empirical evidence suggest that neighborhood exposures change over time for many people and exposures can accumulate to impact population health [7]. Neighborhood effects research is focused on the literal biological incorporation, or embodiment, of the social environments in which people live throughout their life-course [8].

Forced residential displacement due to court-ordered eviction, as well as eviction filings (threat of eviction), are serious public health crises. On average, 2.7 million households are threatened with eviction annually [9]. While housing loss due to court-ordered eviction has deleterious consequences, this underestimates the magnitude and consequence of the threat of eviction for marginalized families [10]. For example, whereas 18.6% of all adult renters are Black, 51.1% of those who experience an eviction filing are Black, and Black women and their children are disproportionately burdened [11].

Structural racism limits the opportunity of Black families to have safe, secure, affordable, and sustainable housing, and increases risk for adverse maternal and infant health outcomes [12]. Calls have been made for more robust research that names which specific place-based experiences and exposures are most salient to groups made vulnerable to embody structural racism [13]. A small and growing body of cross-sectional evidence focuses on neighborhood level eviction rates and risk of preterm birth among Black women [14]. Using birth record data from National Center for Health Statistics (2008–2016) merged to county level eviction rates from the Eviction Lab (2009–2016) [15], Khadka et al. reported a suggestive (though imprecise) association between increased county-level eviction filing rates and a 2.13 percentage point increase in risk of preterm birth for Black women (95% confidence interval: − 0.06, 4.33). Similarly, Harville et al. examined the same ecological data limited to 2015 and reported more precise results suggesting higher odds of PTB for Black women who lived in counties with highest versus lowest eviction filing rates (OR 1.14, 95% CI: 1.06, and 1.23) [16].

However, neighborhood data at the smallest available geographic unit of analysis has been shown to more closely proxy individual experience of neighborhood context. Freedman et al. examined hospital-based birth records from Chicago between 2008 and 2018 linked to data from the Eviction Lab and reported 1.92 times higher odds of PTB for Black women who lived in block groups with highest vs lowest eviction threat (95% CI: 1.26, 2.93), in adjusted models [17]. This study did not have data on individual socioeconomic status, or duration of residence in the current neighborhood, and therefore the results may be confounded by these omitted covariates. On the other hand, Sealy-Jefferson et al., using a cohort from Metro-Detroit, MI (2009–2011), documented a joint association between block group level eviction threat, and marital/cohabiting status and risk of PTB in Black women (relative risk: 1.25, 95% CI: 1.06, 1.47), using linked medical records, surveys, and eviction rate data from the Eviction Lab [18]. Importantly, to our knowledge, no studies to date have taken a life-course approach and incorporated granular, individual-level residential histories in the study of neighborhood threat of eviction and risk of PTB among priority populations.

The current research is informed by several theories including Black feminist thought, which is an important lens through which to center and explore experiences of Black women because it names and therefore makes legible the typically invisible interlocking systems of oppression, as well as liberatory forms of resistance to heteropatriarchal power, and advocacy for human rights [19]. Intersectionality, coined by legal scholar Kimberle’ Crenshaw, is a key element of Black feminist thought, and offers a framework for understanding the unique intersection of racial and gender oppressions experienced by Black women [20]. Reproductive justice, defined as the inextricably linked human rights to have children, not have children, and parent children in safe and healthy environments free from individual and/or state violence [21], is the foundation upon which the current research is built. Our research question was: are neighborhood eviction filing rates, over time, associated with individual PTB risk among Black women? We hypothesized that increasing (compared to steady low) neighborhood threat of eviction over time (from 2 years before to during pregnancy) would be associated with increased risk of PTB.

## Methods

### Study Design

In this novel cohort study, we leverage residential histories reported in surveys and PTB prevalence abstracted from medical records from the Life-course Influences on Fetal Environments Study (LIFE), a retrospective cohort conducted from June 2009 to December 2011 at a suburban hospital in Michigan, USA [22]. The methodological innovation of the current study allows us to overcome the cost and time-intensity of traditional prospective cohort studies and establish temporality between objective exposure variables at two time points, and objectively measured outcome. Specifically, in our data linkage, we reconstructed point- in- time data to answer a change-over-time research question.

### Participants

Potentially eligible participants were identified from hospital logs and approached within 24–48 h after delivery during their hospital stay with an invitation to participate in the research study. Participants were not eligible if they were non-English speaking, had intellectual disabilities, serious cognitive deficits, or evidence of significant mental illness, on the basis of any prior records or history. Inclusion criteria included: delivering a singleton infant, self-identification as African American/Black, and age 18–45 years. In-person interviews were completed during the postpartum hospital stay and medical records were abstracted by trained study staff.

### Procedures

The eviction process exists on a continuum, beginning when a landlord informs a tenant that an eviction warranting event or behavior has occurred and ending when the situation is resolved, or the landlord repossesses the housing; and can last from a few days to an enduring cycle of threatened eviction [10]. For the current study, the exposure was block-group-level eviction filing rates, per 100 renter occupied homes, from the Eviction Lab at Princeton University [15]. We previously linked eviction filing rates from 2007 to 2011 from the Eviction Lab, standardized to reflect Census 2010 boundaries, to the latitude and longitude of self-reported addresses from LIFE study participants from before enrollment (considered preconception, from 2007 to 2009), and at study enrollment (during pregnancy, from 2009 to 2011) [18]. We matched eviction filing rates to LIFE study participant addresses based on year (for example, preconception eviction rates for 2007 were linked to LIFE study participants who enrolled in 2009 and reported their address from 2 years before enrollment).

The outcome was PTB, defined as birth before 37 completed weeks of gestation, abstracted from participant medical records. A hierarchical algorithm was used to categorize the birth as term or preterm (5), with priority given to the gestational age estimate based on early ultrasounds (between 6- and 20-weeks’ gestation), given that this is considered the most valid measure (9).

### Statistical Analysis

**Fig. 1:**
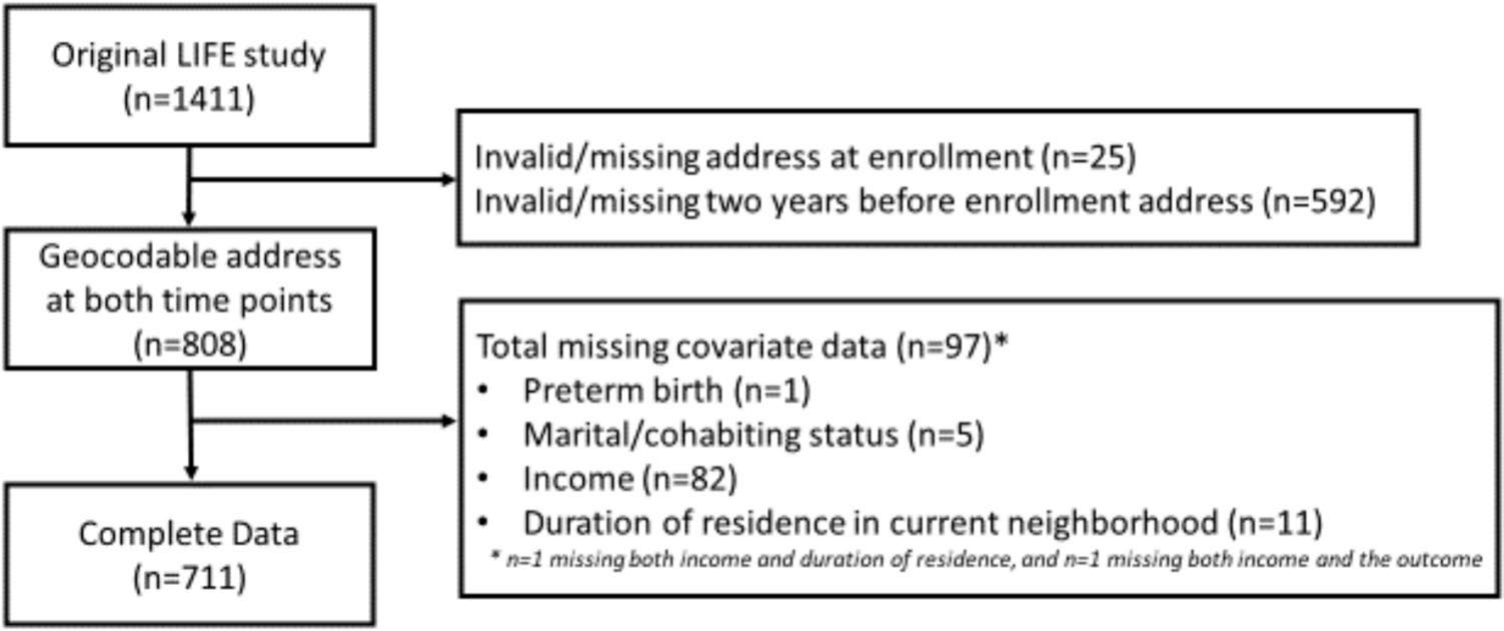
STROBE diagram

The LIFE study included *n* = 1410 participants, which represents 70.5% of those approached for participation (*n* = 1999). The present study included (*n* = 808) LIFE participants who reported their address 2 years before and at study enrollment (Fig. 1). Cut points were determined based on distributions in the sample, and we used univariable and multivariable statistics to describe the data, including chi-square and Wilcoxon rank sum tests which quantified differences in categorical and continuous variables. Missing data on all variables ranged between 0 and 10% (Fig. 1). Since the prevalence of PTB in our sample was > 10%, and we observed little block-group level variation in PTB (which precludes hierarchical modeling), we used modified Poisson regression with robust error variance, to estimate unadjusted (uRR) and adjusted relative risks (aRRs) and 95% confidence intervals (CI) for associations between neighborhood eviction filing trajectories and risk of PTB. Consistent with published research on neighborhood trajectories [23], we dichotomized eviction rates at the median for each time point (low/high), and examined persistent low and high exposure as well as change in exposure to neighborhood eviction filings over time. The trajectory measure had the following 4 preconception/during pregnancy neighborhood combinations: (1) persistent low (low/low), (2) persistent high (high/high), (3) decreasing (high/low), and (4) increasing (low/high). Women who lived in neighborhoods with low/low eviction filing rates were the referent group. To mitigate potential bias due to the non-random nature of where people live, we included predictors of residential selection in our adjusted models, including self-reported age (< 35, 35 +), income (dichotomized at $35,000 per year), marital/cohabiting status (single, married/cohabiting), and educational attainment (≤ 12, > 12 years). To control for neighborhood contextual conditions, we also adjusted for time lived in the neighborhood during pregnancy (dichotomized at 2 years), as well as residential move from preconception to during pregnancy, and a block-group level neighborhood disadvantage index which included a linear composite of nine optimally-weighted, neighborhood-level, socio-economic, and socio-demographic variables from the American community survey for the during pregnancy neighborhood, as described in our prior work [24]. We examined all variables for missing data (Fig. 1) and used listwise deletion. In sensitivity analyses to check the robustness of our results, we repeated our analysis using tertiles of eviction rates at each time point (low/medium/high) which resulted in 9 categories consistent with our prior work [25]. SAS version 9.4 for Windows was used for the statistical analysis (SAS Institute Inc., Cary, NC, USA).

### Role of the Funding Source

The funder of the study had no role in study design, data collection, data analysis, data interpretation, or writing of the manuscript.

## Results

Study participants resided across 534 block groups during pregnancy (median: 1 mean: 1.5 (standard deviation (SD): 1.1), minimum–maximum: 1–7):, and 532 block groups before pregnancy (median: 1, mean: 1.5 (SD: 0.9), minimum–maximum: 1–16. The prevalence of PTB in the sample was 16.2%, and the mean age at enrollment was 27 years (SD: 6.14). Women who were between 20 and 24 years old made up the largest proportion (34%) and those who were 18–19 comprised < 9% of the sample (Table 1). More than half of the participants reported annual income less than $35,000 and being married to or living with the father of their baby. Nearly 70% of the sample had at least 12 years of education, and 41% reported living in their current neighborhood for at least 2 years. Thirty-one percent of participants reported living at the same address before and during pregnancy (*n* = 251). In bivariate analyses, those who were 35 + years old had higher risk of preterm birth (RR: 1.74, 95% CI: 1.05, 2.91) compared to those aged 25–29 years old. There was little evidence of association between marital/cohabitation status, income, time lived in current neighborhood, study enrollment year, or residential move, and risk of preterm birth in bivariate models.

**Table 1:** Socio-demographic characteristics of study participants and bivariate modified Poisson regression results for associations with preterm birth; Life-course Influences on Fetal Environments (LIFE) study, 2009–2011(*n* = 807)

Age	Preterm birth (*N* = 131) *N* (%)	Term birth (*N* = 676) *N* (%)	RR	95% CI
18–19	7 (5.3)	62 (9.2)	0.83	0.38, 1.83
20–24	48 (36.6)	223 (33)	1.45	0.93, 2.26
25–29	26 (19.9)	187 (27.7)	*Referent*	
30–34	27 (20.6)	119 (17.6)	1.52	0.92, 2.49
35 +	23 (17.6)	85 (12.6)	1.74	1.05, 2.91
Marital/cohabiting status				
Single	54 (41.5)	309 (46)	*Referent*	
Married/cohabiting	76 (58.5)	363 (54)	1.16	0.85, 1.60
Education (years)				
≤ 12	39 (27.8)	207 (30.6)	0.97	0.69, 1.36
> 12	92 (70.2)	469 (69.4)	*Referent*	
Income				
< $35,000	74 (60.7)	335 (55.5)	1.19	0.86, 1.67
≥ $35,000	48 (39.3)	269 (44.5)	*Referent*	
Time in during pregnancy neighborhood				
< 24 months	74 (58.3)	392 (58.6)	*Referent*	
≥24 months	53 (41.7)	277 (41.4)	*1.01*	0.73, 1.40
Residential Move From Preconception to During Pregnancy				
Yes	90 (68.7)	467 (69.1)	*1.02*	0.72, 1.42
No	41 (31.3)	209 (30.9)	*Referent*	
Study enrollment year				
2009	20 (15.3)	82 (12.1)	*Referent*	
2010	39 (29.8)	200 (29.6)	*0.83*	0.51, 1.35
2011	72 (55)	394 (58.3)	*0.79*	0.50, 1.23

Abbreviations *RR*, Relative risk; *n*, number; *n*=1 missing preterm birth status

The average neighborhood eviction filing rates before and during pregnancy in the total sample were similar (24–25%) (Table 2). The maximum neighborhood eviction filing rates in some of the neighborhoods our participants lived before and during pregnancy suggests serial eviction filings by landlords. Comparing low and high categories of eviction filing rates over time, the mean and standard deviation were similar in magnitude (Table 2). On average, we observed approximately 26 more eviction filings for the increasing trajectory. The average change for the decreasing trajectory was 23.4 fewer neighborhood eviction filings. The range of eviction filing rates was 0–23.2 before pregnancy, and 25–132 during pregnancy, per 100 renter occupied homes. The lowest proportion of participants were in the decreasing (20.3%) and the highest were in the persistent high trajectory (29.7%) of eviction filing rates (Table 3).

**Table 2:** Neighborhood eviction filing rate categories before and during pregnancy and associated descriptive statistics; Life-course Influences on Fetal Environments (LIFE) Study, 2009–2011 (*n* = 808)

	Before pregnancy			During pregnancy		
Eviction Filing Rate	Total sample	Low (***n*** = 404)	High (***n*** = 404)	Total sample	Low (***n*** = 399)	High (***n*** = 409)
Mean	**24.9**	**12.9**	**36.8**	**24.1**	**13.4**	**38.6**
Median	**23.2**	**14.4**	**32.3**	**25**	**15**	**35**
SD	**16.1**	**6.9**	**13.6**	**16.5**	**7.3**	**13.1**
Range	**0**–**116.7**	**0**–**23.2**	**23.2**–**116.7**	**0**–**132**	**0**–**24**	**25**–**132**

*SD*, standard deviation.

**Table 3:** Distribution of participants in trajectories of neighborhood eviction filing rates before and during pregnancy; Life-course Influences on Fetal Environments (LIFE) Study, 2009–2011 (*n* = 808)

Eviction threat trajectories	Trajectory category	N (%)
Low → low	Persistent low	235 (29.1)
High → high	Persistent high	240 (29.7)
High → low	Decreasing	164 (20.3)
Low → high	Increasing	169 (20.9)

*N*, number; *%*, percent.

Compared to persistent low eviction filing rates over time, persistent high rates were associated with the smallest magnitude of association for risk of PTB (adjusted aRR: 1.11, 95% CI: 0.69, 1.80), followed by decreasing eviction filing rates (aRR: 1.27, 95% CI: 0.77, 2.11). The largest magnitude of association for risk of PTB was for increasing eviction filing rates over time (aRR: 1.68, 95% CI: 1.05, 2.68) (Table 4). Results of sensitivity analyses with a more nuanced 9-category trajectory measure, as described in our prior work [26], were consistent with our primary analyses, especially for the increasing category of eviction filing rates (low/high), although results were less precise (aRR: 1.77, 95% CI: 0.92, 3.38) (Supplement 1).

**Table 4:** Modified Poisson regression results for associations between a 4-category neighborhood eviction filing rate trajectories before and during pregnancy and risk of Preterm Birth among Black women; Life-course Influences on Fetal Environments (LIFE) Study, 2009–2011

Eviction filing rate trajectories		Unadjusted		Adjusted*	
	*n*	RR	95% CI	RR	95% CI
Persistent low	31	Reference			
Persistent high	35	1.11	0.71, 1.74	1.11	0.69, 1.80
Decreasing	29	1.34	0.84, 2.14	1.27	0.77, 2.11
Increasing	36	1.61	1.04, 2.50	1.68	1.05, 2.68

*N*, number with a preterm birth; *RR*, relative risk; *CI*, confidence interval; *n*, number.

*Adjusted for age, income, educational attainment, time lived in current neighborhood, and neighborhood disadvantage index, and residential move from preconception to during pregnancy.

## Discussion

The main finding of this study is that women who lived in neighborhoods with higher eviction threat during pregnancy (compared to before pregnancy), had 68% higher risk of PTB, compared to those who lived in neighborhoods with persistent low eviction threat, in adjusted models (95% CI:1.05, 2.68). Our results are consistent with existing ecologic [16, 27], and cross-sectional studies on the association between neighborhood level eviction threat and PTB [17, 18]. Importantly, our work extends existing literature in that we use within-group analysis of Black women to examine associations between PTB risk and changes over time, of neighborhood eviction threat, during sensitive periods for mothers and their infants.

The proportion of participants was highest in the persistent high trajectory of neighborhood eviction threat (29.7% *n* = 240), which had a combined range of 23.2–132 eviction filings per 100 renter occupied homes. The upper limit of the range of eviction filing rates for the persistent high group suggests serial eviction filings by landlords. Further, the widening of the range of eviction filings over time, indicates increasing threat of eviction during pregnancy. These results are consistent with existing evidence that landlords use repeated eviction filings against the same tenants, not necessarily to repossess the rental unit, but to collect rent and associated penalties [10]. We extend the existing literature by documenting strong associations between increasing eviction filing rates and increased risk of the leading cause of infant death among a priority population. Given the minimal progress in decreasing the population burden of PTBs, despite extensive research, community level toxic stressors resulting from landlord business practices and behavior should be the focus of more research including interventions to decrease predatory landlord behavior, as well as policy change and enforcement of existing policies to protect tenants.

A recent study, using national data from 2007 to 2016, reported that previous estimates had undercounted the number of individuals facing eviction and that 1 in 5 Black adult renters personally experienced an eviction threat [11]. Surprisingly, in our study, the average magnitude of change for the low to high trajectory of eviction filings was about 26 more filings per 100 renter occupied homes. Said differently, in some of the neighborhoods under study, more than 1 in 4 renter occupied homes experienced an eviction threat and/or serial eviction filing against the same residence(s) in a 12-month period. Our research identified a sensitive period of exposure to neighborhood eviction threat that is associated with excess risk of PTB in Black women. These results suggests that single point in time measures likely underestimate the impact of exposures over time. Indeed, we previously reported little evidence of association between during pregnancy neighborhood eviction filing rates and risk of PTB in the overall LIFE study sample. Future research should identify joint associations between other social factors and neighborhood eviction filing trajectories and risk of PTB, as well as potential mediating pathways, to identify novel intervention targets.

Evictions have direct, and spillover effects on the mental and physical health of individuals, including stress for individuals who may not directly experience them [14] by foretelling housing insecurity for neighbors, who may be relatives or friends [27]. Studies show housing instability caused by eviction also impacts physical and social environments of communities through various intersecting mechanisms [28]. For instance, chronic preconception stressors are associated with PTB risk, through biologic mechanisms including but not limited to dysfunction of the hypothalamic-pituitary adrenal axis [29]. Individual stressors as well as those experienced by family, friends, or loved ones have been associated with poor mental health among Black women [30]. Recent evidence suggests increasing neighborhood threat of eviction over time is a key source of severe toxic stress that is associated with increased odds of moderate and serious psychological distress during pregnancy [25].

The unjust and stressful exposure to neighborhood level threat of eviction may help to explain the racial inequity in adverse birth and maternal outcomes. Exposure to this source of toxic stress is unjustly concentrated in Black communities, and likely contributes to intergenerational changes in the biological stress response in this group [29]. Future federal, state, and local legislative regime changes (and enforcement) to protect tenants and their neighbors from the impact of eviction threats by landlords will support the goals of housing and reproductive justice [31]. Hospital systems could support reproductive justice for their patients by screening for and providing resources to mitigate neighborhood level stressors including from the threat of eviction.

### Limitations

The LIFE study did not include data on individual level experience of eviction. Reverse causation cannot be ruled out, given that we do not account for PTB history before study enrollment, as this would attenuate the association we were trying to estimate, since prior PTB is a determinant of future PTB risk. Our analysis includes self-identified women, and we understand that gender is not binary and therefore we cannot generalize our findings to other gender identities. Future research is needed to understand the neighborhood experiences of people across the gender identity spectrum. In our analytic sample, 10% of participants were missing data on income. However, in our prior work using the full LIFE study cohort, with 11% missing on income, results using a sequential regression multiple imputation method (with 20 replicate datasets) were comparable to the associations between neighborhood exposures and risk of PTB in our primary analysis [22]. Furthermore, we adjusted our models for an objective during pregnancy neighborhood disadvantage index, which included a linear composite of nine optimally-weighted, neighborhood-level, socio-economic, and socio-demographic variables from the American community survey, as used in our prior work [24].

### Strengths

Calls to identify the causal effect of eviction threat on adverse birth outcomes have been made [27], though existing available data has largely precluded such study designs. While we cannot make causal inferences, our study extends the existing literature, in that- using novel data linkages- we establish the temporal order of neighborhood eviction threat over time precedes risk of PTB. We also adjusted our models for self- reported duration of residence in the during pregnancy neighborhood, moves over time, and individual and neighborhood socioeconomic status, which is an improvement on existing research. Our within group analysis allows us to move beyond disparity documentation, to identify a novel, policy-relevant, and modifiable risk factor for PTB among a population group that is made vulnerable by an understudied and highly prevalent manifestation of structural racism.

### Summary

Without targeted policy and practice interventions, eviction threat is likely to continue at crisis levels globally, especially in countries with weak welfare states and market based housing systems [28]. Future research should identify effect measure modifiers as well as mechanisms of the association between neighborhood eviction threat and risk of PTB, as well as document the breadth, scope, and nature of the spill-over effects within and across communities.

## Supplementary Information

Below is the link to the electronic supplementary material.

ESM 1 (DOCX. 15.4 KB)

## Data Availability

LIFE Study data are available by request to the LIFE Study principal investigator (Dr. Dawn P. Misra).
