# Supplementary material for: Neighborhood Threat of Eviction over Time and Risk of Preterm Birth in Black American Women
Source: J Racial Ethn Health Disparities. 2025 May 13;13(4):2905–12. doi: 10.1007/s40615-025-02465-y (PMC13346131; doi:10.1007/s40615-025-02465-y)
Supplement: Supplementary file 1 — (DOCX. 15.4 KB) [file 40615_2025_2465_MOESM1_ESM.docx]

**Supplementary Information**

**Supplemental Table 1**. Modified Poisson regression results for associations between a 9-category neighborhood eviction filing rate trajectories before and during pregnancy and risk of Preterm Birth among Black women; Life-course Influences on Fetal Environments Study, 2009-2011.

| **9-category Eviction Filing Rate Trajectories** | **Relative Risk (95% Confidence Interval)** |
| --- | --- |
| Low-Medium | 1.31 (0.66, 2.59) |
| Low-High | 1.77 (0.92, 3.38) |
| Medium-Low | 1.01 (0.49, 2.10) |
| Medium-Medium | 1.00 (0.52, 1.93) |
| Medium-High | 1.39 (0.71, 2.70) |
| High-Low | 0.54 (0.19, 1.58) |
| High-Medium | 1.45 (0.75, 2.80) |
| High-High | 0.85 (0.42, 1.71) |
